# Graph Neural Networks-Based Prediction of Drug Gene Interactions of RTK-VEGF4 Receptor Family in Periodontal Regeneration

**DOI:** 10.4317/jced.61880

**Published:** 2024-12-01

**Authors:** Pradeep Kumar Yadalam, Francisco T. Barbosa, Prabhu Manickam Natarajan, Carlos M. Ardila

**Affiliations:** 1 Department of Periodontics, Saveetha Dental College and Hospital, Saveetha Institute of Medical and Technical Sciences, Saveetha University, Chennai- 600077, TamilNadu; 2 Private practice, Spalentorweg 38,4051 Basel, Switzerland; 3 Assistant Professor in Periodontics, Department of Clinical Sciences, Center of Medical and Bio-allied Health Sciences and Research, College of Dentistry, Ajman University, Ajman, UAE; 4 DDS. Titular Professor. Universidad de Antioquia U de A, Medellín, Colombia. Biomedical Stomatology Research Group, Universidad de Antioquia U de A, Medellín, Colombia

## Abstract

**Background:**

The RTK-VEGF4 receptor family, which includes VEGFR-1, VEGFR-2, and VEGFR-3, plays a crucial role in tissue regeneration by promoting angiogenesis, the formation of new blood vessels, and recruiting stem cells and immune cells. Machine learning, particularly graph neural networks (GNNs), has shown high accuracy in predicting these interactions. This study aims to predict drug-gene interactions of the RTK-VEGF4 receptor family in periodontal regeneration using graph neural networks.

**Material and Methods:**

The study utilized a dataset comprising 19,154 drug-gene interactions to analyze the relationships between drugs and protein-coding genes. The dataset was split into training and testing sets, with 80% of the data used for training and 20% for testing. Cytoscape, an open-source software platform, was employed to visualize and analyze the drug-gene interaction network, and CytoHubba, a plugin, was used to identify highly connected nodes. Topological measures were applied to determine the influence and importance of each node. GNNs were used to manage the complex relationships and dependencies within the graphs.

**Results:**

The drug-gene interaction network, comprising 815 nodes and 13,436 edges, was found to be complex and highly interconnected. It was divided into 11 components, displaying low density and heterogeneity, indicative of a sparse structure. The GNN model achieved 97% accuracy in predicting interaction types, including single protein interactions and protein complex groups.

**Conclusions:**

The study demonstrates that graph neural networks outperform traditional machine learning methods in predicting drug-gene interactions within the RTK-VEGF protein family in periodontal regeneration, highlighting their potential in advancing therapeutic strategies and drug discovery.

** Key words:**Graph neural networks; drug-gene interactions; RTK-VEGF4 protein family: periodontal regeneration.

## Introduction

The RTK-VEGF4 receptor family, comprising VEGFR-1, VEGFR-2, and VEGFR-3, is vital for tissue regeneration (1,2). VEGFR-1 facilitates angiogenesis by forming new blood vessels and recruiting stem cells and immune cells. VEGFR-2 promotes new blood vessel growth and wound healing through angiogenesis, while VEGFR-3 is crucial for lymphangiogenesis, forming new lymphatic vessels essential for efficient tissue repair. Manipulating these receptors can enhance tissue regeneration and repair outcomes (3).

Periodontal regeneration involves activating the RTK-VEGF4 receptor family to promote angiogenesis. VEGFR-1, expressed on endothelial cells, stimulates new blood vessel formation, providing oxygen, nutrients, and immune cells to regenerate damaged teeth. VEGFR-2, the primary receptor in VEGF-induced angiogenesis, supports endothelial cell survival and vascular permeability, facilitating regeneration (4). VEGFR-3 regulates lymphangiogenesis, aiding in the clearance of inflammatory molecules and promoting a more efficient healing response. Vascular Endothelial Growth Factors (VEGFs) regulate angiogenesis by binding to VEGFRs and coreceptors. VEGFRs, along with VEGF-A and C, play roles in lymphatic system formation, with VEGFR-3 and its ligands having both angiogenic and lymphangiogenic effects (5).

The VEGF receptor family is essential for periodontal regeneration by promoting angiogenesis, vasculogenesis, and lymphangiogenesis. These processes are crucial for forming new blood vessels and delivering nutrients and oxygen to tissues. VEGF-A, a member of the VEGF ligand family, is upregulated in periodontal wound healing, binding to VEGFR-2 (6,7), the primary VEGF receptor in endothelial cells. Other VEGF receptors, such as VEGFR-1 and VEGFR-3, also contribute to periodontal regeneration. Therapeutic strategies targeting these receptors may enhance periodontal regeneration.

Drug-gene interactions (8,9) involve the interplay between drugs and specific genes in the biological pathways targeted by the drug. For the RTK-VEGF4 receptor family, drugs can modulate the activity or expression of these receptors, influencing angiogenesis and periodontal regeneration. Therapeutic agents targeting this pathway include anti-angiogenic drugs, such as monoclonal antibodies or tyrosine kinase inhibitors, that inhibit VEGF signaling and limit angiogenesis (10,11). Conversely, gene therapy approaches aim to enhance RTK-VEGF4 receptor family signaling by introducing genes encoding these receptors or their ligands, potentially promoting tissue regeneration in periodontal disease. Understanding drug-gene interactions within the RTK-VEGF4 receptor family is crucial for developing effective therapeutic strategies to enhance periodontal regeneration outcomes.

By collecting data, extracting features, and training models, machine learning accurately predicts drug-gene interactions, which is crucial for drug discovery, personalized treatment, and understanding the molecular basis of diseases. Graph neural networks (GNNs) (12) are a popular method for predicting drug-gene interactions. These networks handle graph-structured data, representing entities like drugs and genes as nodes and modeling their interactions using graph edges. The GNN model updates hidden representations by aggregating information from neighboring nodes and can be trained using a supervised learning approach with known drug-gene interactions as labels. GNNs have shown promising results in predicting drug-gene interactions, although they require careful feature engineering and can be computationally expensive.

Our study aims to use Graph Neural Network Prediction to analyze Drug-Gene Interactions of the RTK-VEGF4 Receptor Family in periodontal regeneration.

## Material and Methods

-Dataset Preparation:

The study utilized a dataset of 19,154 drug-gene interactions sourced from https://www.probes-drugs.org/ to analyze drug-protein gene interactions (13). Annotation and preprocessing steps involved assigning information and labels to nodes, classifying them as drugs or genes, and defining interactions as edges. Additional features such as biochemical activity and drug-gene interaction type were incorporated. Outliers were removed to ensure data quality. The dataset was then divided into training and testing sets, with 80% allocated for training and 20% for testing.

-Cytoscape and CytoHubba:

Cytoscape (14), an open-source software platform, was used to visualize and analyze the drug-gene interaction network. Nodes in the dataset represented drugs and genes, while edges depicted their interactions. Cytoscape provided various layout algorithms for effective visualization. Built-in tools and plugins were utilized to calculate network properties, identify key genes, and perform topological analysis. CytoHubba, a plugin, was employed to identify highly connected nodes. Topological measures determined the influence and importance of each node. Customizable visualizations highlighted key genes and drugs, and results were exported as network images, Tables, and statistical measures.

-Graph Neural Network Architecture:

A Graph Neural Network (GNN) (15) consists of multiple layers, each performing message passing and node update operations. This iterative process refines node representations by incorporating information from neighboring nodes. The input layer is a graph with nodes that have associated features. Message passing involves nodes exchanging messages with their neighbors, which are then aggregated to represent the node’s neighborhood. The aggregation function determines how these messages are combined. Node update involves updating the node’s representation based on the aggregated information.

GNNs are neural network models designed to operate on graph-structured data, making them popular for handling complex relationships and dependencies in graphs. GNNs start with an input layer that takes node features as input and perform a message-passing operation to exchange information between nodes. This process includes message computation and aggregation, where nodes compute and aggregate messages from their neighbors. The update function then updates the node’s representation based on the aggregated messages. GNN architectures typically have multiple layers of message passing and node update operations, allowing nodes to refine their representations using global information. The output layer produces the required output for the task, which can be customized for desired predictions or classifications.

## Results

**Figure 1:**
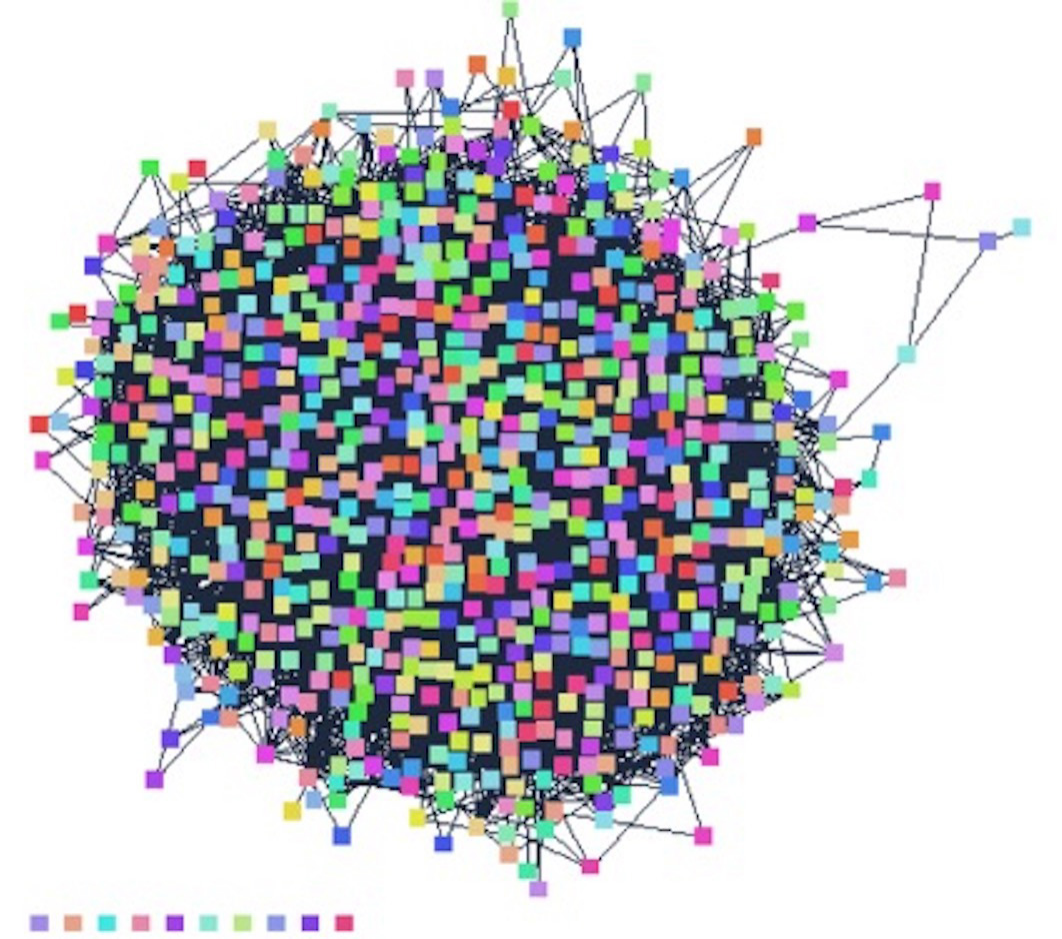
Network Interactome of Genes.

The drug-gene network is composed of 815 nodes and 13,436 edges, forming a complex and highly interconnected structure. Its intricate connectivity, efficient information transfer, and diverse distribution of node degrees contribute to this complexity. The network is segmented into 11 connected components, representing distinct subnetworks (Fig. 1). The network’s low density and heterogeneity suggest a sparsely connected structure, with node degrees and centralization scores indicating an absence of centralized control.

**Figure 2:**
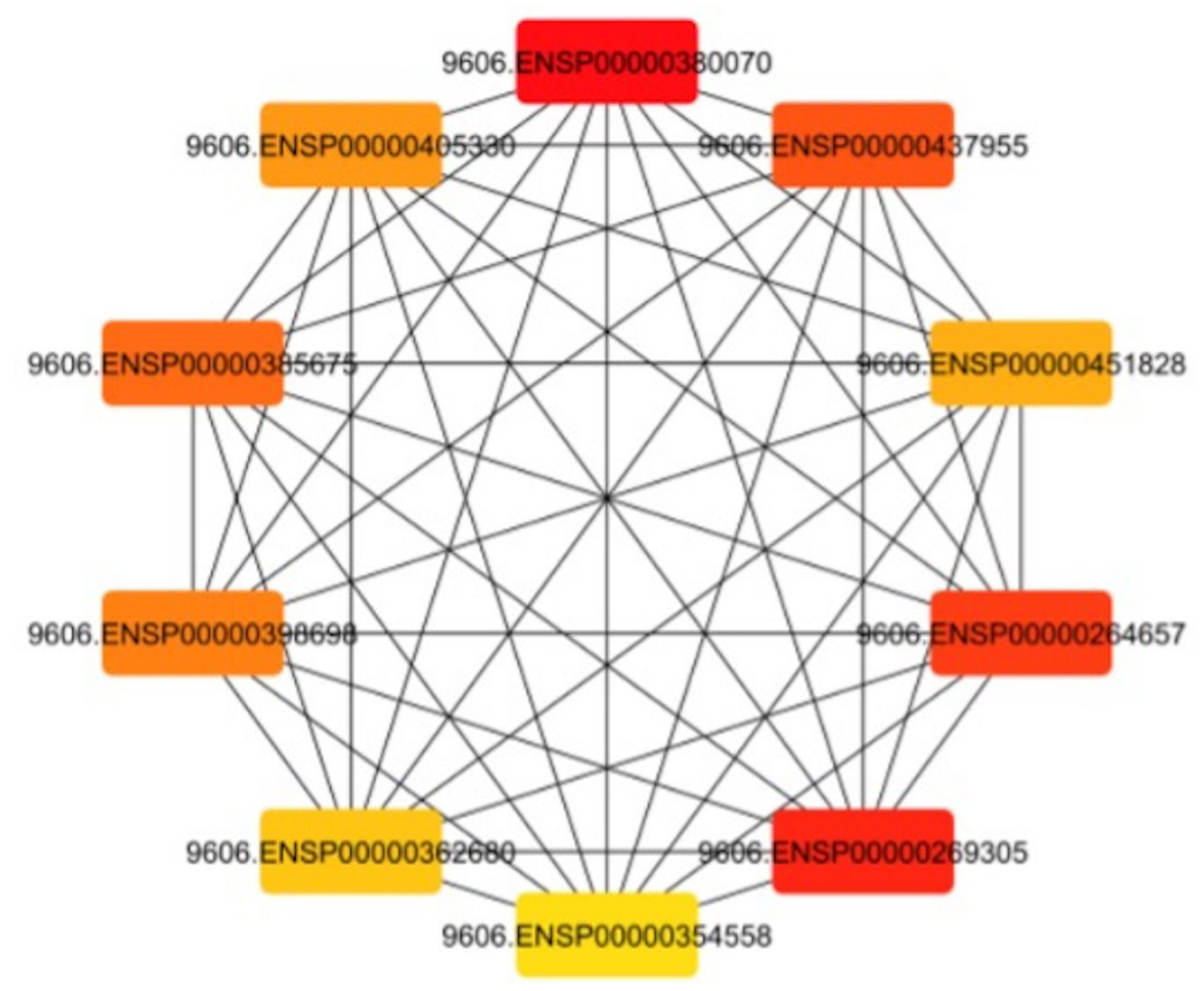
Identification of the Top Ten Hub Genes Using the CytoHubba Plugin.

The top ten hub genes identified in the RTK-VEGF receptor analysis using CytoHubba are STAT3, IL6, HIF1A, TP53, MTOR, AKT1, GAPDH, ESR1, TNF, and SRC (Fig. 2). The GNN model achieved 97% accuracy in predicting interaction types, including single protein edges, protein-protein interactions, and protein complex groups.

**Figure 3:**
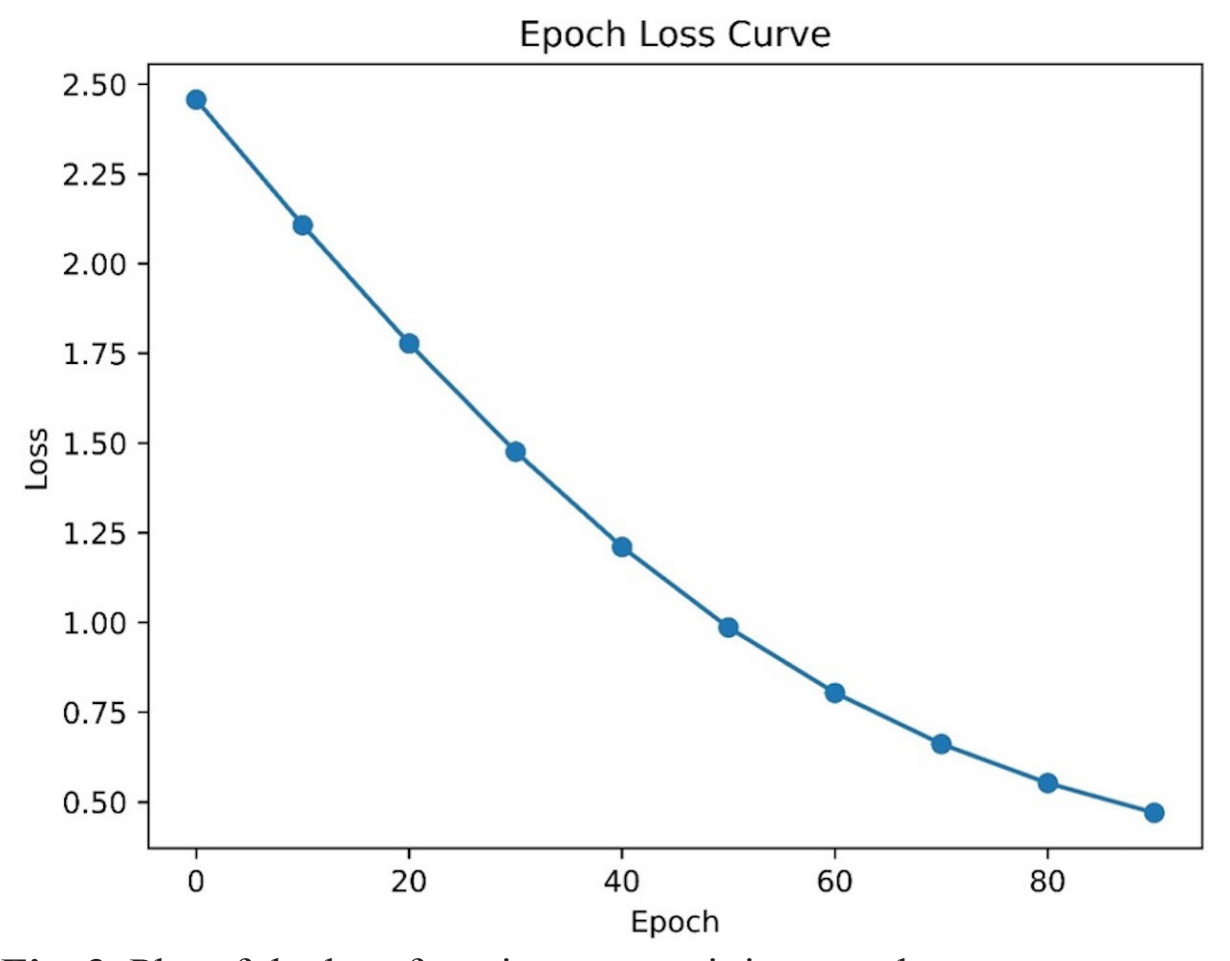
Plot of the loss function over training epochs.

The ROC curve and epoch loss curve offer further insights into the model’s performance (Fig. 3). Figure 3 shows a plot of the loss function over training epochs. The loss decreases as the number of epochs increases, indicating that the model is learning and improving. The plot is titled “Epoch Loss Curve” and has labels for both the x and y axes. The x-axis represents the number of epochs, and the y-axis represents the loss value.

**Figure 4:**
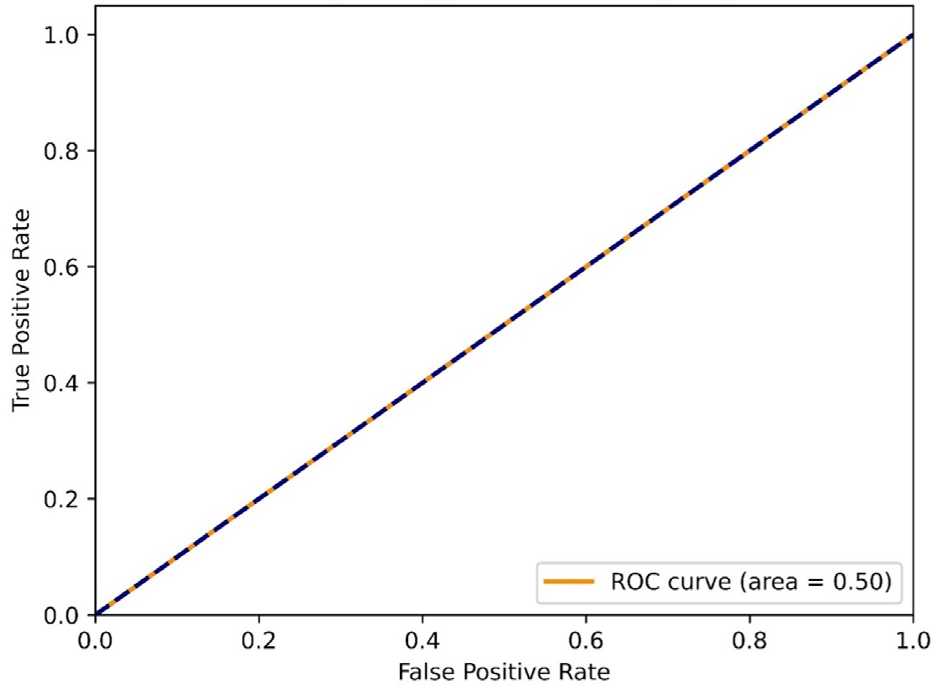
ROC curve.

The Receiver Operating Characteristic (ROC) curve is a graphical tool used to assess the performance of a binary classification model (Fig. 4). By comparing ROC curves, one can identify which model performs better. The Area Under the Curve (AUC) provides a summary of the model’s overall performance, where a value of 1 indicates perfect performance, and a value of 0.5 suggests no discriminative power. The interpretation of the ROC curve is based on its closeness to the top-left corner or its position relative to the diagonal line.

## Discussion

Periodontitis is a widespread condition with significant global impact, making it essential to understand its causes and potential treatments. Studies have shown that VEGF expression is higher in patients with periodontitis compared to healthy controls, indicating that VEGF may play a role in the disease’s pathogenesis and could serve as a potential therapeutic target (1,2,5).

The top hub gene in this drug-gene interaction network is STAT3 (16), a protein that transmits signals from VEGF-A, leading to changes in vascular smooth muscle cells (VSMCs). VEGF-A is known to promote the growth of endothelial cells and new blood vessels through angiogenesis. Previous research has shown that ligature-induced periodontitis in rats causes inflammatory responses, cognitive impairments, and abnormal APP processing, with STAT3 pathway activation contributing to inflammation. Another study indicated that human gingival fibroblasts (HGFs) contribute to periodontitis development by increasing IL-6 production, a response to pathogens and cytokines, and interacting with fibroblasts and macrophages through VEGF activation (7). Synthetic (+)-terrein has been found to be non-cytotoxic and inhibits IL-6-induced protein phosphorylation and VEGF secretion, suggesting its potential anti-inflammatory properties in combating inflammatory disease progression.

Higher levels of HIF-1α and VEGF have been observed in periodontal disease, suggesting that the HIF-1α pathway might be activated in advanced disease stages, possibly influenced by bacterial endotoxins and inflammatory cytokines (4,17). Gingival crevicular fluid levels were found to be affected by disease status, indicating that the TNF-α/HIF-1α/VEGF pathway might play a role in periodontal disease pathogenesis. One study showed no significant difference in P53 expression between groups, suggesting that chronic periodontitis may not notably impact P53 expression, and that apoptosis changes related to P53 in this condition are minimal (5,17). These hub genes may play a role in the RTK-VEGF protein receptor family in regeneration.

The GNN model demonstrated a 97% accuracy in predicting drug-gene interactions, though it does not account for the trade-off between true positive and false positive rates. The ROC curve plots the true positive rate (TPR) against the false positive rate (FPR) at various thresholds, while accuracy considers all classes and misclassifications equally. Discrepancies between the two can arise from class imbalance, threshold selection, and varying costs of errors.

Future directions for GNNs include increasing dataset size to maintain high accuracy across different data distributions, external validation to assess generalizability, robustness testing to evaluate reliability against noise and disruptions, and improving interpretability to explain VEGF drug-gene predictions (18-20). However, limitations include potential biases in training data, the trade-offs between false positives and negatives, and the importance of feature representation for accurate drug-gene interactions. Additionally, computational and scalability issues may require optimizations and efficient implementation strategies to reduce training and inference times (16,21). Future research should focus on developing methods to interpret and explain GNN predictions, particularly for drug-gene interactions in the RTK-VEGF protein family.

## Conclusions

This study highlights the effectiveness of Graph Neural Networks in predicting drug-gene interactions within the RTK-VEGF protein family, surpassing traditional machine learning methods. Factors such as dataset size, training duration, feature availability, and model complexity influenced the performance of the GNN. However, the study faced limitations, including the dataset’s incomplete representation of the entire RTK-VEGF protein family and potential biases in the training data. Future research is necessary to optimize GNN models and evaluate their reliability in the context of personalized medicine for periodontal regeneration.

## Data Availability

The datasets used and/or analyzed during the current study are available from the corresponding author.
